# P-1784. Reduction of bacterial resistance in isolates from nosocomial infections through a program of antibiotic stewardship based on a local mobile application in a hospital in Mexico City; 10 years of success

**DOI:** 10.1093/ofid/ofae631.1947

**Published:** 2025-01-29

**Authors:** Paulo Castañeda-Méndez, José Luis Castillo-Álvarez, Esperanza Aleman Aguilar, Griselda Medina-Montaño, L U I S E Soto-Ramirez, Daniel Aguilar-Zapata, Javier Reyes Mar

**Affiliations:** Hospital Medica Sur, Tlalpan, Distrito Federal, Mexico; Médica Sur, Tlalpan, Distrito Federal, Mexico; Medica Sur, Mexico City, Distrito Federal, Mexico; Medica Sur, Mexico City, Distrito Federal, Mexico; Hospital Medica Sur, Tlalpan, Distrito Federal, Mexico; Hospital Medica Sur, Tlalpan, Distrito Federal, Mexico; Hospital Medica Sur, Tlalpan, Distrito Federal, Mexico

## Abstract

**Background:**

Hospital-acquired infections caused by multidrug-resistant microorganisms (MDR-HAI) are associated with high healthcare costs, prolonged hospital stays, and concerning morbidity and mortality. The prevalence of MDR-HAI has increased worldwide as a result of the COVID-19 pandemic. In this context, antimicrobial stewardship programs (ASP) have become more relevant.

**Figure:**
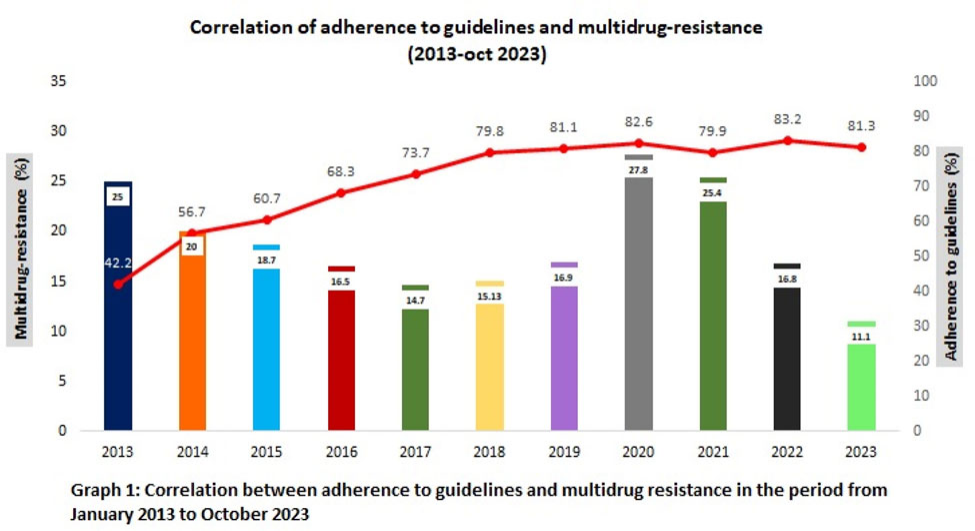

**Methods:**

A mobile application was implemented for hospital staff to guide optimal antibiotic use. This application is based on local resistance patterns for major nosocomial microorganisms, to provide treatment recommendations (initial regimen based on suspicion, adjustment according to microbiological results, antibiotic discontinuation, etc.). Descriptive statistics were performed.

**Results:**

In 2013, before the implementation of the application, the compliance rate was 25% for hospital-acquired infections and 42.2% adherence to guidelines. After the introduction of the application, compliance in 2014 was 56.7%, with a reported 20% MDR-HAI rate. In 2015, the usage was 60.7% and MDR-HAI rate was 18%. In 2016, compliance was 68.7% and MDR-HAI rate was 16%. In 2017, compliance was 73.7% and MDR-HAI rate was 14%. In 2018, the prevalence of MDR-HAI was 15%, while the usage of local guidelines was 78.7%. In 2019, compliance was 81.1% and MDR-HAI rate was 16.9%. Considering the COVID-19 pandemic (starting in 2020) as a period in which there was a significant increase in the presence of MDR-HAI worldwide, thanks to the implementation of the application, the percentage of MDR-HAI decreased from 27.8% to 11.1% in 2023 (Image 1). The number of different types of infections (VAP, HAP, UTI, ICS, BSI) also decreased by following the guidelines recommendations.

**Conclusion:**

These programs are necessary as part of the healthcare program to control the morbidity and complications of MDR-HAI. Simple and user-friendly electronic applications, such as the one implemented in our hospital, have led to increased compliance and a decrease in bacterial resistance of infections acquired in our institution.

**Disclosures:**

**All Authors**: No reported disclosures

